# Secondary Dengue Infection Complicated by Hemophagocytic Lymphohistiocytosis: A Case Report

**DOI:** 10.1155/crdi/9208878

**Published:** 2025-07-30

**Authors:** Dominique D. Davis, Saffett Guleryuz, Yehuda Galili, Pablo A. Bejarano

**Affiliations:** ^1^Department of Pharmacy, Department of Hematology and Oncology, Cleveland Clinic Florida, Weston, Florida, USA; ^2^Department of Hematology and Oncology, Cleveland Clinic Florida, Weston, Florida, USA; ^3^Department of Pathology and Laboratory Medicine, Cleveland Clinic Florida, Weston, Florida, USA

## Abstract

Hemophagocytic lymphohistiocytosis is a fatal hyperinflammatory disorder in which CD8+ cytotoxic T-cells, natural killer cells, and macrophages destroy hematopoietic cells and vital organs. Viral infections, such as Epstein–Barr virus, are known to cause secondary hemophagocytic lymphohistiocytosis in adult patients. However, despite its rarity, dengue virus has been identified to potentially cause hemophagocytic syndrome, which is associated with significant mortality and morbidity. Herein, we present a case report of a 52-year-old male patient who presented with fevers, worsening non-bloody copious diarrhea, excessive fatigue, and nausea and vomiting. He was known to have sickle cell trait. A diagnosis of hemophagocytic lymphohistiocytosis was confirmed with a liver biopsy, accompanied by elevated ferritin levels (33,539 ng/mL), IL-2R levels (5944.2 pg/mL), thrombocytopenia (49 k/μL), anemia (hemoglobin and mean corpuscular volume of 7.3 g/dL and 77.3 fL), and elevated bilirubin (total bilirubin of 3.2 mg/dL). In addition, elevated IgG and IgM antibodies determined reinfection with dengue virus. The administration of dexamethasone, etoposide, and additional supportive medications was initiated. Despite all efforts, the patient's neurological status declined, and the patient died. In this case, dengue-induced hemophagocytic lymphohistiocytosis is a worrisome and challenging diagnostic condition, primarily due to the similarities between the symptoms of hemophagocytic lymphohistiocytosis and those of dengue hemorrhagic fever. Treatment delay may be an inevitable consequence. Differentiating between dengue hemorrhagic fever and dengue-induced hemophagocytic lymphohistiocytosis requires evaluating clinical, laboratory, and biopsy findings. The role of the sickle cell trait is unknown in the presentation.

## 1. Introduction

Hemophagocytic lymphohistiocytosis (HLH) is a life-threatening hyperinflammatory disorder in which immune cells destroy hematopoietic cells and vital organs. The cells involved in the pathogenesis are uncontrolled activated cytotoxic T-cells, natural killer (NK) cells, and histiocytes [1]. Persistent activation of these cells leads to the secretion of large amounts of inflammatory cytokines, including interferon (IFN)-γ, interleukin (IL)-1, IL-2, IL-6, and IL-18, and tumor necrosis factor-α (TNF-α) [1, 2]. The excessive cytokine production contributes to multiorgan failure and tissue destruction [3]. HLH is primarily a pediatric disease driven by a mutated genetic predisposition. However, an inappropriate host response to infections, malignancies, and immunodeficiency contributes to secondary etiology in adults.

The activation of secondary HLH is typically related to viral, fungal, or bacterial infections. Common pathogens such as Epstein–Barr virus (EBV), *Mycobacterium tuberculosis, Staphylococcus aureus*, cytomegalovirus (CMV), and human immunodeficiency virus (HIV) are proven causes of developing HLH [4, 5]. Recently acquired hemophagocytic syndrome following dengue infection has been recognized as a potentially fatal complication characterized by activated lymphocytes, jaundice, and hepatosplenomegaly [6]. Dengue virus (DENV) is a mosquito-transmitted virus that contains four genetically distinct serotypes (DENV 1–4) [7]. Unfortunately, infection with any DENV serotype results in long-term homotypic immunity, accompanied by a brief period of heterotypic immunity against other serotypes. Thus, dengue patients with secondary infections may develop more severe life-threatening complications than the initial infection, including organ impairment, capillary leak syndrome, and severe bleeding.

Due to the similarity of symptoms between HLH and severe DENV fever, a definitive diagnosis is complicated, and implementation of supportive treatment can be delayed. Practical consideration for assessing the signs of HLH involves using clinical and laboratory findings to determine extreme inflammation. Valuable labs such as biomarkers (e.g., CD25) and complete blood count (CBC) with differential, ferritin, and triglyceride levels are more specific indicators for an accurate diagnosis. In addition, a biopsy of the bone marrow and involved organs is required to assess the presence of hemophagocytosis [8].

Once a diagnosis is confirmed, treatment is required immediately to improve survival. Addressing the underlying disease is essential in the overall treatment concept; however, if ineffective, the mainstay treatment with immunosuppressive (dexamethasone 10 mg/m^2^) and chemotherapeutic (etoposide 150 mg/m^2^) agents is preferred for HLH. Both therapies assist with suppressing the hyperinflammatory response of immune cells and inducing apoptosis of cytotoxic T-cells. Other treatment modalities, including intravenous immunoglobulin (IVIG), anakinra, and alemtuzumab are effective and increase overall survival [9]. This case report involves a patient with sickle cell trait (SCT) who was fatally reinfected with DENV and complicated by liver failure, septic shock, respiratory failure, neurologic effects, and HLH. High ferritin levels, thrombocytopenia, anemia, and a liver biopsy confirmed evidence of acquired HLH. Treatment was initiated with corticosteroids, chemotherapy, and other supportive agents. Informed consent and permission to publish this case report were obtained from the patient's family.

## 2. Case Presentation

A 52-year-old male with a past medical history notable for hypertension, hyperlipidemia, type-2 diabetes mellitus, and SCT presented to the medical intensive care unit (MICU) from an outside facility for the assessment and management of acute liver failure of an unknown etiology. Upon admission, the patient was intubated, sedated, and hemodynamically stable. Family and social history were unknown at the time of admission. The patient was under a hematologist's care for chronic leukocytosis and lymphocytosis related to SCT.

Outside hospital records unveiled that the patient recently traveled to Jamaica and reported gastrointestinal symptoms with diarrhea upon return. He admitted experiencing several mosquito bites during his visit and denied unusual food consumption. A few days later, the patient sought medical attention due to fevers (*T*_max_ 39.1°C), worsening non-bloody copious diarrhea (up to 10 episodes), excessive fatigue, nausea, and vomiting. Notably, the patient denied any sick contacts, and accompanying family members did not exhibit signs of illness. Home medications included atorvastatin 20 mg daily, losartan 50 mg daily, and metformin 1000 mg twice daily.

Laboratory investigations from the outside hospital revealed thrombocytopenia (platelets of 49 k/μL), borderline microcytic anemia without overt sign of bleeding (hemoglobin and mean corpuscular volume of 7.3 g/dL and 77.3 fL), renal insufficiency (serum creatinine of 3.90 mg/dL), significant transaminitis (AST and ALT of 724 IU/L and 379 IU/L), elevated bilirubin (total bilirubin of 3.2 mg/dL), rhabdomyolysis (creatinine kinase of 2373 units/L), acetaminophen level was < 5 μg/mL (10–30 μg/mL), and labile blood pressure. Intravenous fluids were started with 3 L of normal saline and 150 mEq of sodium bicarbonate. Subsequently, the patient's hemodynamic and mental status deteriorated, leading to intubation following respiratory failure with consolidation in both lung bases concerning multifocal pneumonia. Imaging studies revealed fatty liver disease with hepatomegaly, contracted gallbladder with gallstones, and a shrunken spleen. Infectious and rheumatological workups revealed positive dengue immunoglobulin G (IgG) and negative for complete viral hepatitis, malaria, HIV, and SARS-CoV-2 (COVID-19), and stool studies including *Clostridioides difficile* infection were unremarkable.

The patient arrived at our facility with ventilation requirements (positive end-expiratory pressure [PEEP] of 10, FiO2: 65%, and O2 saturation: 94%) and management for acute liver failure. Initial assessments upon arrival indicated derangements in the biochemistry and hematology panel (Table 1). Liver ultrasound revealed hepatic steatosis, and CT scans demonstrated mild to moderate ascending colon wall thickening suggestive of nonspecific colitis, cholelithiasis, and small-volume ascites. The patient was promptly admitted to the MICU for the management of acute liver failure of an unknown etiology. The patient was immediately started on continuous renal replacement therapy (CRRT) and received red blood cells and platelet transfusions. No occult signs of bleeding were noted.

Given the patient's recent travel history, presentation of symptoms, positive dengue IgG, and a significant rise in the number of dengue fever cases reported in Jamaica during this time, it was suspected that the patient may have severe dengue fever due to reactivation. The peripheral blood smear showed pancytopenia and microcytic anemia without polychromasia. Hemolysis workup was normal for reticulocyte count and haptoglobin levels. A transjugular liver biopsy with pressure measurements due to thrombocytopenia was performed to assess further decompensation. Immunoserology tests were negative for various viral infections; however, reinfection with DENV was confirmed as both IgG and IgM antibodies were positive Table 2. Therefore, severe dengue fever with plasma leakage (small-volume ascites) was the most likely diagnosis. The patient was given 500 mL of lactated Ringer and albumin (5%). The possibility of dengue shock syndrome was considered, as the patient became hypotensive despite fluid resuscitation.

**Figure 1:**
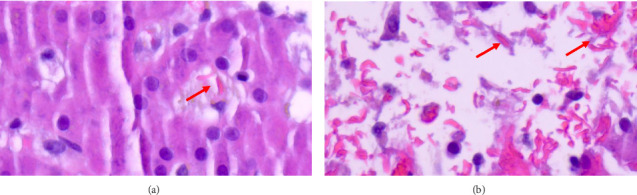
Liver biopsy. A sickled red blood cell is engulfed by a sinusoidal Kupffer cell that also contains a brown hemosiderin pigment.

HLH was suspected due to the presence of multiple suggestive features, including high ferritin levels (33,539 ng/mL), thrombocytopenia (platelets of 53 k/μL), two lineages of cytopenia (hemoglobin of 7.7 g/dL), hypertriglyceridemia (310 mg/dL), and high serum IL-2R of (5944.2 pg/mL). However, hypofibrinogenemia and splenomegaly were not present during the initial assessment. Given the patient's critical condition with shock, treatment for HLH was deferred until a bone marrow biopsy was performed. The bone marrow biopsy showed extensive necrosis of the marrow and was suboptimal for assessment. However, the liver biopsy showed features of HLH, sinusoidal histiocytes engulfing red blood cells, and sickled red blood cells (Figure 1). Imaging showed no abnormality or bleeding in the retroperitoneum. Also, the right upper quadrant ultrasound was performed for possible evaluation of liver hematoma post-biopsy; however, no bleeding was seen. Sickle cell crisis was excluded since it is unexpected in individuals with the sickle trait. His initial HScore was 193 with an 80%–88% probability of hemophagocytic syndrome.

Considering the patients' high-risk complications and death from the disease and treatment, the benefits of starting therapy outweigh the risks of fungemia since the patient was on effective antifungal therapy. HLH-specific therapy, comprising of dexamethasone 30 mg daily and etoposide 112 mg/m^2^ on Day 8 and 75 mg/m^2^ on Day 11 of hospitalization (dose reductions due to liver dysfunction), was initiated. Supportive care and infection prophylaxis were started with acyclovir 200 mg every 12 h, levofloxacin 750 mg every 48 h, and fluconazole 400 mg daily. An early improvement was seen as the patient's vasopressor requirements decreased, arterial pH improved, and the patient remained afebrile.

**Figure 2:**
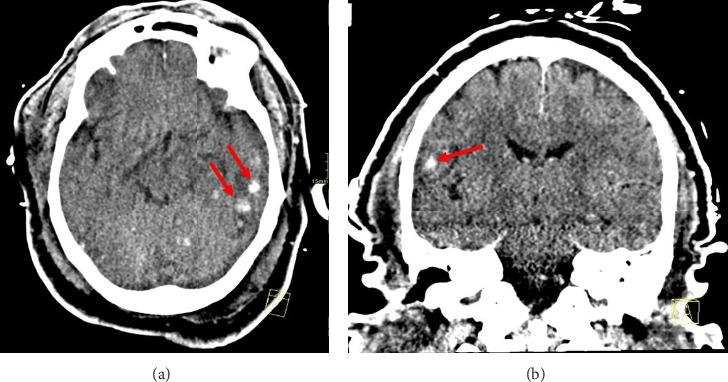
CT scan of the head. (a) Computed tomography (CT) head without contrast showing multiple peripherally located subcentimeter hemorrhages. (b) Computed tomography (CT) head with IV showing parenchymal hemorrhages.

Unfortunately, on Day 13 of admission, the patient's neurologic status worsened. Evidence on CT scans revealed multifocal intraparenchymal hemorrhages with vasogenic edema (Figure 2). Fungemia due to *Candida tropicalis* persisted despite therapy, and repeated blood cultures were positive for *E. coli* bacteremia. The patient's antifungal coverage was switched to micafungin, as *Candida tropicalis* was susceptible to both azoles and echinocandins. Despite maximal vasopressor support, 2 L of normal saline, and escalation of antibiotics, the patient began to deteriorate clinically. The clinical team's discussion with the family reached a consensus for changing the code status to do not resuscitate (DNR). The patient developed pulseless electrical activity (PEA) and expired.

## 3. Discussion

This report describes a fatal case of a 52-year-old male reinfected with dengue fever. Complications of the virus were liver failure, septic shock, respiratory failure, neurologic effects, and HLH. The diagnosis of HLH was made based on increased ferritin and triglyceride levels and confirmed by a liver biopsy. This case is unique in the sense that our patient had a preexisting hematologic disorder of SCT. Although individuals with the trait are typically asymptomatic, and the condition is considered benign, a few studies and case reports suggest a correlation between SCT and complications associated with various viral infections. One systematic review and meta-analysis analyzed COVID-19 outcomes (mortality and hospitalization) in individuals with sickle cell disease (SCD) or SCT compared to individuals without SCD or SCT [10]. Eight studies adjusted for confounders demonstrated that patients with SCD/SCT were shown to be at increased risk of death (OR: 1.86; 95% CI: 1.30–2.66; *p* = 0.0007) compared to those without SCD/SCT [10]. The adjusted confounders analysis for hospitalization revealed higher rates for the SCD (OR: 5.44; 95% CI: 1.55–19.13; *p* = 0.008; *I*^2^ = 97%) and the SCT groups (OR: 1.31; 95% CI: 1.10–1.55; *p* = 0.002; *I*^2^ = 0) compared to the non-SCD/SCT population [10]. Although SCT is less severe than SCD, complications from viral infections may be exacerbated in these patients.

Dengue-associated HLH has been well-reported in children and adults. Presenting symptoms of a primary infection resolve in a week with supportive care. However, subsequent infections with different serotypes are more apparent and develop into severe DENV infections, such as dengue hemorrhagic fever (DHF) and dengue shock syndrome [11]. Our patient's progression from mild to severe symptoms may be attributed to antibody-dependent enhancement (ADE) in dengue. ADE is a unique phenomenon where suboptimal antibodies, mainly IgG, that target viral proteins from one serotype are ineffective against other serotypes and exhibit poor functioning [12]. These antibodies are insufficient at neutralizing DENV, resulting in viral entry into immune cells and promoting viral replication and proinflammatory responses [12]. This pathological link may contribute to the rapid clinical decline commonly seen in severe dengue infections.

HLH is uncommon in DENV cases, and hemophagocytic syndrome is not typically suspected. However, it is essential to differentiate between the diseases. Even though dengue causes prolonged or recurrent fevers, splenomegaly and hemophagocytosis are mainly seen in HLH, which was evident in our patient. Therefore, comprehending the physiology of HLH as an inflammatory disease rather than a distinct ailment can be difficult. Standardized diagnostic tools, such as HLH-2004 diagnostic criteria and the HScore in conjunction with clinical judgment, serve as objective measures to identify HLH. Both tests evaluate fever, splenomegaly, cytopenia (≥ 2 cell lines), hypertriglyceridemia or hypofibrinogenemia, hyperferritinemia, and hemophagocytosis in the bone marrow, spleen, or lymph nodes [9, 13]. For our patient, his liver biopsy showed features of HLH, and his HScore was 193, with an 80%–88% probability of hemophagocytic syndrome. Although the HScore can be helpful, its use has limitations as it has not been validated in a critical care population. Despite its use in practice, the interpretation of an HScore for secondary HLH may be inaccurate due to the complexity of the syndrome.

In treating dengue fever complicated by HLH, administering a cytotoxic agent with immunosuppressants is the mainstay of therapy. Although etoposide-containing regimens are reserved for children, they have proven to be effective in adult outcomes [14, 15]. One retrospective study containing a subgroup analysis using eight patients with dengue-induced HLH demonstrated that 4 (80%) of the five patients who received directed therapy with dexamethasone±etoposide responded to treatment, and 7 out of 8 patients were still alive, with a median survival of 18.41 months [16]. In another case report, a patient presented with acute liver failure complicated by dengue and HLH received dexamethasone (10 mg/m^2^) daily and oral etoposide (150 mg/m^2^) twice a week [17]. Blood counts and liver function improvements were noticed in the first month, and cytopenia, organomegaly, and ascites were resolved. In other cases, the initiation of corticosteroids alone has been shown to be effective in treating dengue-induced HLH. In two case reports, both patients presented with fevers, headaches, fatigue, and end-organ damage with an HScore of 176 and 200 [18]. With high clinical suspicion of HLH, intravenous dexamethasone 10 mg/m^2^ was initiated daily. By Day 9, improvement in liver enzymes and recovery of blood counts were noted. Early recognition and diagnosis of dengue-associated HLH and prompt intervention could have contributed to the favorable outcome in both patients [18].

Other treatment modalities have been introduced and are known to be effective for dengue-associated HLH, depending on the presentation of symptoms. In one case report, anakinra was administered to an 11-year-old girl who presented with dengue-induced HLH with a progressively worsening clinical condition, having developed multiorgan dysfunction syndrome requiring intubation [19]. IVIG and methylprednisolone were administered, but the patient's clinical condition worsened. Anakinra was initiated, and improvement was noted within 48 h. Although IVIG was ineffective in the aforementioned study, other studies have demonstrated its efficacy and reduction in mortality for patients. The use of IVIG in combination with intravenous steroids also proved effective in 72 patients diagnosed with secondary HLH, as symptoms resolved in 76% of patients, and 82.5% of patients were still alive six months after diagnosis [20]. The use of less cytotoxic agents has been demonstrated to be safe and improve overall survival; however, more research is required to determine the therapeutic benefits in those with infections related to secondary HLH. Etoposide was chosen as our patient's primary treatment based on clinical guidelines and a review of the literature.

## 4. Conclusion

In this case, preexisting comorbid conditions, recent travels to dengue fever endemic areas, and the onset of multiorgan failure all contributed to the difficult diagnosis and treatment of HLH despite intensive interventions. Understanding the pathophysiology of HLH and exploring novel treatment approaches remains crucial for improving outcomes in similar clinical situations.

## Figures and Tables

**Table 1:** Trend of laboratory investigations.

Labs with reference range	Day 1^∗^	Day 2	Day 3	Day 4^∗∗^
White blood cell count (3.70–11.00 k/μL)	6.58	4.94	2.68	1.73
Red blood cell count (4.20–6.00 m/μL)	2.82	2.67	1.92	2.65
Hemoglobin (13.0–17.0 g/dL)	8	7.6	5.4	9.1
Hematocrit (39.0%–51.0%)	21.6	20.6	14.7	22.4
Platelets (150–400 k/μL)	27	34	27	53
MCV (80–100 fL)	76.6	78.3	76.6	79.2
MCH (26.0–34.0 pg)	28.4	28.4	28.1	29.1
CRP (< 0.5 mg/dL)	12.2			
ESR (0–15 mm/hr)	12	—	—	—
Procalcitonin (< 0.10 ng/mL)	19.14			
Sodium (136–144 mmol/L)	143	140	141	135
Potassium (3.7–5.1 mmol/L)	4.2	—	4.7	5.3
Chloride (97–105 mmol/L)	92	92	99	100
Carbon dioxide (22–30 mmol/L)	32	23	20	21
Calcium (8.5–10.2 mg/dL)	7.2	7.3	7.3	7.1
Serum creatinine (0.73–1.22 mg/dL)	6.85	5.47	3.48	2.61
Magnesium (1.7–2.3 mg/dL)	2.1	2.2	2.4	2.5
Phosphorous (2.7–4.8 mg/dL)	6.5	3.8	3.9	4.7
ALT (10–54 U/L)	262	—	—	66
AST (14–40 U/L)	387	—	—	—
Total bilirubin (0.2–1.3 mg/dL)	2	2.6	2.8	2.3
Ferritin (30.3–565.7 ng/mL)	—	—	—	33,539
Triglyceride (< 150 mg/dL)	—	—	—	310
Fibrinogen (200–400 mg/dL)	363	287	481	503
INR (0.9–1.3)	1.2	1.3	1.4	1.5
aPTT (23.0–32.4 s)	—	33.0	33.0	34.0

*Note:* ALT: alanine aminotransferase; AST: aspartate aminotransferase.

Abbreviations: aPTT = activated partial thromboplastin time; CRP = C-reactive protein; ESR = erythrocyte sedimentation rate; INR = international normalized ratio; MCH = mean corpuscular hemoglobin; MCV = mean corpuscular volume.

^∗^Labs obtained on admission after being transferred from an outside facility.

^∗∗^Labs obtained concerning hemophagocytic lymphohistiocytosis.

**Table 2:** Microbiology panel.

Dengue fever IgG antibodies (< 1.64)	11.77
Dengue fever IgM antibodies (< 1.64)	2.95
Epstein–Barr virus (EBV)	No serological evidence of EBV infection
Hepatitis A, B, and C panel	Negative
Cytomegalovirus (CMV)	Negative
Human T-lymphotropic virus (HTLV)	Negative
Herpes simplex virus (HSV) PCR	Negative
Human immunodeficiency virus (HIV)	Non-reactive
Leptospira antibody IGM	Negative
Syphilis	Negative
Blood parasite	Negative

## Data Availability

Data sharing is not applicable to this article as no new data were created or analyzed in this study.
